# Criterion-Referenced Standards of Handgrip Strength for Identifying the Presence of Hypertension in Croatian Older Adults

**DOI:** 10.3390/jcm12196408

**Published:** 2023-10-08

**Authors:** Peter Sagat

**Affiliations:** Sport Sciences and Diagnostics Research Group, GSD/Health and Physical Education Department, Prince Sultan University, Riyadh 11586, Saudi Arabia; sagat@psu.edu.sa

**Keywords:** muscle strength, handgrip dynamometer, elderly, cut-points, high blood pressure

## Abstract

Background: It is well known that muscular fitness has been associated with hypertension. However, it is less known which cut-off values of muscular fitness may predict the presence of hypertension. The main purpose of this study was to establish criterion-referenced standards of muscular fitness to define the presence of hypertension in Croatian older adults. Methods: In this cross-sectional study, we recruited men and women over 60 years of age. Muscular fitness was assessed by handgrip strength and normalized by height squared. Hypertension was defined as having systolic blood pressure ≥130 mm/Hg or diastolic blood pressure ≥80 mm/Hg. Results: In older men, the optimal cut-point of muscular fitness in defining hypertension was 15.4 kg/m^2^. The area under the curve (AUC) was 0.85 (96% CI 0.77 to 0.92, *p* < 0.001). In older women, the optimal cut-point was 11.8 kg/m^2^, with an AUC of 0.84 (95% CI 0.80 to 0.89, *p* < 0.001). Men and women with cut-points of < 15.4 kg/m^2^ and < 11.8 kg/m^2^ were 11.8 (OR = 11.8, 95% CI 4.3 to 32.4, *p* < 0.001) and 10.6 (OR = 10.6, 95% CI 5.7 to 19.7, *p* < 0.001) times more likely to be diagnosed with hypertension. Conclusions: Our newly developed cut-points of muscular fitness assessed by the handgrip strength and normalized by height squared have satisfactory predictive validity properties in detecting men and women aged 60-81 years with hypertension.

## 1. Background

The number of people aged 60 years and older is rapidly increasing worldwide [1]. Estimates suggest that the prevalence of older people will exceed the number of young people by 2050 [2]. With the increased age, most older people are suffering from developing health diseases and disability [3]. Among many, hypertension has been considered one of the most prevalent chronic diseases [4]. It adds to the burden of cardiovascular diseases, stroke, kidney failure, and premature mortality [5]. Despite the strategies that have been implemented to prevent and manage hypertension [6], the mortality trend for hypertension continues to increase annually [7].

Aging is associated with a decline in muscle strength [8]. Its loss is usually accompanied with a substantial decrease in muscle mass [8] and lower levels of physical activity [9]. Lower levels of muscle strength may result in sarcopenia and poorer functional ability [10].

The assessment of muscle strength involves a variety of methods and tools that measure voluntary movements related to strength, including the force of knee extension, hip flexion, and handgrip [11]. Handgrip strength is a simple and low-cost test for evaluation of individual’s muscle strength [11,12], especially for the elderly population [13].

Evidence shows that handgrip strength has been inversely associated with cardiovascular disease [14], whereas hypertension represents a well-known cardiovascular risk factor [15]. Although previous studies have shown that greater handgrip strength is associated with lower risk of hypertension [16,17,18,19], there is no consensus regarding cut-points for identification of risk for hypertension. An effort has been made to establish handgrip strength cut-points in older adults to detect sarcopenia [20,21], muscle weakness [22], mobility limitations [23], and diabetes [24]. The most commonly used cut-point in the literature defines grip strength less than 26 kg and 32 kg in men and less than 16 kg and 20 kg in women as ‘weak’ and ‘intermediate’ [22]. From a public health perspective, the inclusion of handgrip strength measurements in health surveillance systems is important, because lower handgrip strength has been consistently associated with mortality and cardiovascular disease [25]. Thus, older adults seem to be an appropriate population for monitoring fitness, in order to offer interventions for those with high blood pressure values. Given the importance of muscular strength for future health-related outcomes, cut-points to determine older adults at an increased risk of hypertension are required and could be used as a starting point for promoting greater muscular strength and lowering hypertension risk.

Therefore, the main purpose of this study was to establish criterion-referenced standards of muscular fitness to define hypertension in older adults. Such evidence would give a deeper insight into the development of national-based normative values used in clinical, nursing, and independent living settings.

## 2. Methods

### 2.1. Study Participants

This study used the same sample as the previous cross-sectional study conducted on Croatian older adults [26]. In brief, the participants were part of a single rehabilitation center with a general aim to investigate the lifestyle habits of apparently healthy older adults, who went through annual systematic check-ups from 2020 to 2022. At the first stage, a convenient sample of 843 men and women aged ≥60 years old were recruited. According to the project’s aims and hypotheses, the inclusion criteria for entering the study were as follows: (i) being without chronic diseases, which included chronic heart disease, rheumatic arthritis, chronic kidney disease, stroke, cancer, and chronic obstructive pulmonary disease, (ii) the absence of a serious physical or mental illness, and (iii) matching all the study variables tested for. On the other hand, the exclusion criteria were as follows: (i) having acute or chronic locomotor or psychiatric diseases at the time of measurement, (ii) not matching all the variables tested for in order to be included in further analyses due to absence or personal reasons. Of the initial sample, 180 (53 men and 127 women) did not match all the parameters necessary for the study, and 20 (8 men and 12 women) had acute or chronic locomotor or psychiatric diseases. After re-analysis, 643 men and women matched all the study variable measurements and were included in the study (260 men and 383 women). Before data collection started, all participants were informed about the aim, hypotheses, and methodology of the study. The participants were ensured confidentiality and informed that their participation was voluntary, and that they had the right to withdraw at any time. All participants have read and signed the informed consent forms. We followed the methods of the principles of the Declaration of Helsinki [27], and the Ethical Committee of The Home of War Veterans approved the study (Ethical code number: 2022/4).

### 2.2. Blood Pressure Measurement

Blood pressure was measured at one time point. Each participant was instructed to be calm for 5 min in a sitting position with no vigorous exercise prior to testing. Blood pressure was assessed using a standard mercury sphygmomanometer blood pressure cuff. The cuff was placed on the right mid-arm at the same level as the heart. The systolic blood pressure value was noted at the first Korotkoff sound and the diastolic blood pressure was measured at the fifth Korotkoff sound [28]. The average systolic and diastolic blood pressure were taken. During the testing, the practitioner was not dressed as a doctor, in order to simulate a home environment and discard the ‘white-coat hypertension syndrome’. The presence of hypertension was defined as having systolic blood pressure ≥130 mm/Hg or diastolic blood pressure ≥80 mm/Hg [28]. Although such classification has been mainly used for American populations, instead of the European cut-off value of ≥140/90 mm/Hg [29], previous evidence suggests using the 130/80 mm/Hg threshold for both patients with cardiovascular risk and the whole population [30]. Also, the guidelines from America and Europe recommend modifying lifestyle for individuals with a blood pressure of 130/80 mm/Hg [29].

### 2.3. Handgrip Strength Measurement

To assess handgrip strength, we used a Jamar Plus* + Digital Hand Dynamometer (Sammons Preston Inc., Bolingbrook, IL, USA). The device was calibrated by the manufacturer, with a precision of 0.1 kg. The protocol for measuring handgrip strength was devised by the American Society of Hand Therapists [31]. In brief, the participant was placed in a seated position with their shoulder rotated and adducted in a neutral position, forearm in neutral position, elbow flexed at 90°, and wrist between 0 and 30° of dorsiflexion. Each participant conducted the measurement three times with the non-dominant hand [29]. Out of three measurements, the best one was recorded and used in further analyses. For the purpose of this study, handgrip strength was normalized by height squared, since previous studies have suggested using body height as the best body size variable for performing allometric normalization of handgrip strength among older adults [32,33].

### 2.4. Anthropometric Measurement

Body height and weight were objectively measured using Seca portable stadiometer and digital scale with a precision of 0.1 cm and 0.1 kg. Body height was measured in bare or stocking feet standing upright against a stadiometer and body weight was measured while wearing light clothes with no shoes. Body mass index was calculated (weight [kg]/height [m]^2^). Waist circumference was measured using anthropometric tape placed horizontally midway between the lower rib margin and the iliac crest at the end of normal expiration, while the participant was standing still [34]. To assess fat mass and fat-free-mass, we used bioelectrical impedance analysis (Omron BF500 Body Composition Monitor, Omron Medizintechnik, Vernon Hills, IL, USA). The device uses eight electrodes and pre-programmed equations to determine fat mass and fat-free-mass estimations. The participant was required to stand on metal footpads barefoot and grasp a pair of electrodes fixed on a handle with arms extended in front of the chest [35].

### 2.5. Statistical Analysis

The statistical analyses were conducted using Statistical Packages for Social Sciences version 23 (SPSS Inc., Chicago, IL, USA). Descriptive statistics were calculated for all variables. Continuous variables were presented as means and standard deviations (SD). Categorical variables were described with unweighted sample counts and weighted percentages. Sex differences were examined using Student *t*-test for independent samples. The magnitude of the differences between the sexes in each variable was calculated using Cohen’s *D* effect size (ES). According to Hopkins et al. [36], ES was classified as trivial (<0.2), small (0.2–0.6), moderate (0.6–1.2), large (1.2–2.0), very large (>2.0), and extremely large (>4.0). We used receiver operating characteristics (ROC) curves quantified by the area under the curve (AUC) to determine a discriminatory ability of handgrip strength to predict hypertension. ROC curves analyses are specialized for demonstrating discriminatory power of a certain diagnostic test, where the curve of the test skews closer to the upper left corner [37]. The AUC is described as the diagnostic power of a test. A classification of the AUC is the following: (i) 0.55–0.62 (small), (ii) 0.63–0.71 (moderate), and (iii) >0.71 (large) [38]. Sensitivity and specificity characteristics were calculated and presented as the number of participants (*N*) and percentages (%). To examine the probability of hypertension based on newly developed cut-points, a set of logistic regression analyses with odds ratios (OR) and 95% confidence intervals (95% CI) were performed. In addition to logistic regression analyses, we used Spearman’s rank correlation analyses to examine the correlation coefficients between handgrip strength and hypertension, stratified by sex and adjusted for age. Two-sided *p*-values were used, and significance was set at α < 0.05.

## 3. Results

Table 1 shows the basic descriptive statistics of the study participants. Older men were taller, heavier, and had higher body mass index values compared with women. Older women had lower waist circumference and waist-to-height ratio values, but higher fat mass compared with men. No significant differences in systolic and diastolic blood pressures between sexes were found. Also, a similar percentage of men and women were classified as ‘hypertensive’. Compared with women, men exhibited larger handgrip strength values.

**Figure 1:**
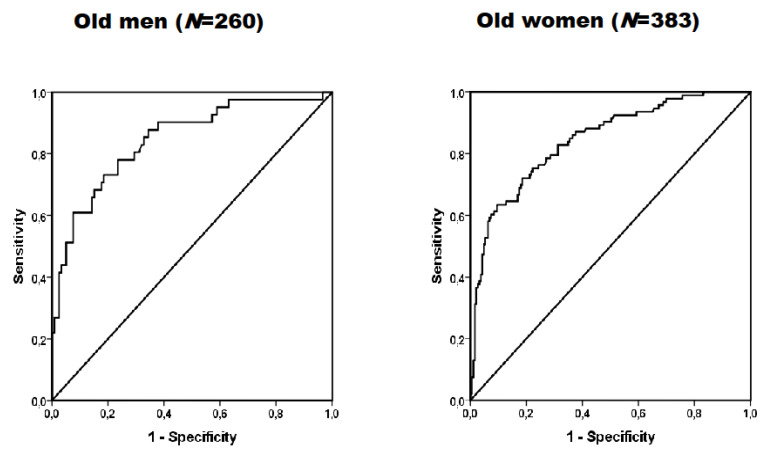
ROC curves of handgrip strength to detect hypertension in older men and women.

Figure 1 shows the ROC curves of handgrip strength to detect hypertension in older men and women. The diagnostic properties of handgrip strength used to detect hypertension are presented in Table 2. For both men and women, handgrip strength showed a significant predictive capacity to detect hypertension (AUCs > 0.80). Men with lower handgrip strength determined by the ROC were (<15.4 kg/m^2^) were almost 12 times (OR = 11.8, 95% CI 4.3 to 32.4, *p* < 0.001) more likely to have hypertension. In women, lower handgrip strength (<11.8 kg/m^2^) was associated with 10.6 (OR = 10.6, 95% CI 5.7 to 19.7, *p* < 0.001) more likelihood of having hypertension. The Spearman’s rank correlation analysis between handgrip strength and hypertension showed moderate correlation coefficients for older men (*r* = −0.50, *p* < 0.001) and older women (*r* = −0.54, *p* < 0.001). Of note, we performed the correlations between handgrip strength and fat-free mass and between fat-free mass and hypertension and found only small correlations for older men (*r* = 0.15, *p* = 0.063 and *r* = −0.09, *p* = 0.255) and older women (*r* = 0.16, *p* = 0.008 and *r* = −0.14, *p* = 0.024).

Sensitivity and specificity properties for newly developed cut-points of handgrip strength are presented in Table 3. Sensitivity for detecting hypertension was strong in both men (87.8%) and women (82.8%), while specificity ranged from 31.1% in women to 37.8% in men.

## 4. Discussion

The main purpose of this study was to establish criterion-referenced standards of muscular fitness to define hypertension in Croatian older adults. The key findings of the study are the following: (i) handgrip strength normalized by height squared has an excellent discriminatory ability to detect older men and women with hypertension, (ii) older men and women with low handgrip strength are 11.8 and 10.6 times more likely to be diagnosed with hypertension, and (iii) sensitivity analysis shows good classification for both sexes.

This is the first study using handgrip strength normalized by height squared to detect the risk of hypertension in older adults. Previous criterion-referenced standards among older adults have been established to detect sarcopenia [20,21], muscle weakness [22], mobility limitations [23], and diabetes [24]. For example, a study by Bahat et al. [20] showed that the cut-off thresholds for handgrip strength to detect sarcopenia were 32 kg and 22 kg for older men and women. Another study on the same topic presented somewhat smaller handgrip strength values; the cut-points for detecting sarcopenia were 27 kg and 16 kg for men and women, respectively [21]. Similar thresholds have been defined for muscle weakness, where older men and women categorized as being ‘weak’ had an absolute handgrip strength <26 kg and <16 kg [22]. For mobility limitations, the overall handgrip strength cut-points in men and women aged 55 years and older were 37 kg and 21 kg [23]. Finally, handgrip strength cut-points normalized by body weight ranged between 0.49 in women and 0.68 in men aged 50–80 years and was used to predict diabetes [24]. For comparison, absolute cut-points for handgrip strength and cut-points for handgrip strength normalized by weight in our study were 49.8 kg and 0.62 in men and 31.5 kg and 0.45 in women. The discrepancy between the cut-points has come from different methodologies. For example, previous studies have used absolute handgrip strength values to determine clinically significant cut-point thresholds [20,21,22,23]. It should be noted that handgrip strength can be confounded by body size, generating the aforementioned discrepancies [39]. This would imply that larger individuals would exhibit greater results when absolute strength values are considered, compared with smaller ones [32]. Our justification for normalizing handgrip strength by height squared is based on the most often used presumption of ‘geometric similarity’, where muscle force should be proportional to body height squared [40]. Of note, AUCs for handgrip strength normalized for body weight, body mass index, fat mass, and waist circumference showed lower values compared with height squared (AUCs ranging from 0.75 to 0.81 in men and from 0.75 to 0.79 in women). The second mechanism is related to different magnitudes of associations between handgrip strength with other health-related outcomes. Lower levels of handgrip strength have been consistently associated with a higher incidence of chronic diseases [20,21,22,23,24]. Although we found strong associations between handgrip strength and the risk of hypertension, some of the previous studies have shown no association [41] or even a positive association between handgrip strength and hypertension [42]. Peripheral vascular resistance increases with chronological age, leading to reduced sympatholysis and elevated sympathetic tone [43].

Although evidence from cross-sectional studies suggests that lower handgrip strength is associated with hypertension [16,17,18], the mechanism underlying longitudinal associations is still relatively unclear [19,41]. Some findings highlight the effect of regression dilution bias, where time fluctuations and changes in handgrip strength may result in the underestimation of the true association between handgrip strength and hypertension [44]. Indeed, handgrip strength represents a reliable and valid method for assessing an individual’s hand motor abilities in both clinical and epidemiological practices [11]. Although pharmacological therapy for treating hypertension has been widely used, patients with hypertension taking hypertensive drugs are more prone to side effects like renal damage, cerebral hypoperfusion, and syncope [45]. Physical exercise is in general a well-proven method for reducing both resting and ambulatory blood pressure, irrespective of the type of physical training (aerobic, resistance, or combined training) [46]. Moreover, a significant body of evidence has revealed that isometric handgrip training interventions may reduce resting blood pressure [47] due to vessel endothelium-dependent dilation, oxidative stress, and autonomic regulation of heart rate and blood pressure [48]. It has been highlighted that physical exercise has comparable and even superior effects for reducing blood pressure, compared with other healthy lifestyle options, positioning physical exercise as a critical component of first-line treatment for high blood pressure [46].

The strengths of this study include a relatively large sample size of older men and women. However, a convenient recruitment of the participants may not allow us to generalizable the findings of the study to other similar sex and age populations. Establishing the cut-off points of adjusted handgrip strength by height to detect hypertension is the novelty added to the literature. Most previous research have used absolute handgrip strength, however [20,21,22,23,24], the absolute value cannot adequately discriminate between individuals with different body size measures.

This study is not without limitations. First, by using a cross-sectional design, we were unable to establish causal associations and cut-points based on longitudinal data. Second, to be able to diagnose hypertension, three measures on three separate days are required. By examining both systolic and diastolic blood pressure at one time point, it is possible that the prevalence of hypertension was overestimated. A 24 h ambulatory blood pressure control is of great significance for the diagnosis of hypertension. Third, we did not include potential residual covariates, such as diet and genetic factors, which might have influenced cut-points and the strength of the associations. Fourth, it should be noted that handgrip strength was only obtained from a non-dominant hand, failing to execute the association between handgrip strength asymmetry and hypertension. Larger handgrip strength asymmetry has been associated with future risk of neurodegenerative and locomotor disorders [49,50]. Finally, we did not have a control group to which the measurement protocol and obtained findings could have been compared to.

## 5. Conclusions

In summary, the analysis used an established approach to identify hypertension cut-points associated with muscle strength defined by handgrip strength. The findings of this study are the first step in screening muscle strength among older adults and detecting individuals with a higher risk of hypertension. The findings also highlight the utility of normalizing handgrip strength by height squared. Allometric normalization is mandatory for handgrip strength in order to remove the body size effect over performance, and height should be used instead of other anthropometric measures, such as body mass, body mass index, and fat mass [32,33]. Therefore, health-related and epidemiological practitioners may use handgrip strength relative to height to accurately detect older adults with hypertension.

## Figures and Tables

**Table 1:** Basic descriptive statistics of the study participants, stratified by sex (*N* = 643).

Study Variables	Men (*N* = 260)	Women (*N* = 383)	ES	*p* for Sex
	**Mean (SD)**	**Mean (SD)**		
**Age (years)**	67.4 (5.5)	66.9 (5.2)	0.09	0.160
**Height (cm)**	172.9 (5.0)	161.1 (6.0)	2.14	<0.001
**Weight (kg)**	84.0 (10.3)	70.0 (12.1)	1.25	<0.001
**Body mass index (kg/m^2^)**	27.7 (3.3)	26.9 (4.2)	0.21	0.027
**Waist circumference (cm)**	100.1 (9.3)	90.5 (11.6)	0.91	<0.001
**Waist-to-height ratio**	0.58 (0.1)	0.56 (0.1)	0.20	0.033
**Fat mass (%)**	31.2 (7.0)	38.2 (6.6)	1.03	<0.001
**Fat-free mass (%)**	70.1 (4.6)	61.8 (6.6)	1.46	<0.001
**Systolic blood pressure (mm/Hg)**	142.5 (17.1)	140.5 (19.6)	0.11	0.104
**Diastolic blood pressure (mm/Hg)**	86.5 (10.1)	86.9 (10.1)	0.04	0.700
**Hypertension (% of ‘yes’)**	74.5	67.0	/	0.107
**Handgrip strength (kg)**	46.9 (7.6)	30.5 (5.3)	2.50	<0.001

*p* < 0.05.

**Table 2:** Receiver operating curve cut-offs for handgrip strength to predict hypertension, stratified by sex (*N* = 643).

Study Variables	Hypertension (Systolic Blood Pressure ≥130 mm/Hg or Diastolic Blood Pressure ≥80 mm/Hg)				
**Handgrip Strength (kg/m^2^)**	**AUC**	**95% CI**	**Std. Error**	***p*-Value**	**Cut-Off Point**
Older men (*N* = 260)	0.85	0.77 to 0.92	0.04	<0.001	15.4 kg/m^2^
Older women (*N* = 383)	0.84	0.80 to 0.89	0.03	<0.001	11.8 kg/m^2^

*p* < 0.05.

**Table 3:** Sensitivity and specificity for handgrip strength cut-offs and hypertension, stratified by sex (*N* = 643).

Older Men (*N* = 260)	Hypertension (Systolic Blood Pressure ≥130 mm/Hg or Diastolic Blood Pressure ≥80 mm/Hg)			
**Handgrip Strength (kg/m^2^)**	**‘No’, *N* (%)**	**‘Yes’, *N* (%)**	**Chi-Square Test**	***p*-Value**
<15.4 kg/m^2^	5 (12.2%)	74 (62.2%)		
≥15.4 kg/m^2^	36 (87.8%)	45 (37.8%)	30.5	<0.001
**Older women (*N* = 383)**				
**Handgrip strength (kg/m^2^)**				
<11.8 kg/m^2^	16 (17.2%)	130 (68.8%)		
≥11.8 kg/m^2^	77 (82.8%)	59 (31.1%)	66.4	<0.001

Denotes using percentages (%).

## Data Availability

The datasets used and/or analyzed during the current study are available from the author on reasonable request.
